# Defining and researching the concept of resilience in LGBT+ later life: Findings from a mixed study systematic review

**DOI:** 10.1371/journal.pone.0277384

**Published:** 2022-11-11

**Authors:** Anže Jurček, Brian Keogh, Greg Sheaf, Trish Hafford-Letchfield, Agnes Higgins

**Affiliations:** 1 Faculty of Social Work, University of Ljubljana, Topniška ulica, Ljubljana, Slovenia; 2 School of Nursing and Midwifery, Trinity College Dublin, Dublin, Ireland; 3 The Library of Trinity College Dublin, Dublin, Ireland; 4 School of Social Work & Social Policy, University of Strathclyde Glasgow, Glasgow, Scotland; National Institute of Public Health: Instituto Nacional de Salud Publica, MEXICO

## Abstract

Within the literature, resilience is described as either a trait, an outcome or a process and no universal definition exists. A growing body of research shows that older LGBT+ adults show signs of resilience despite facing multiple inequalities that negatively impact their health and social wellbeing. The aim of this review was to examine how resilience is defined in LGBT+ ageing research and how it is studied. A mixed-study systematic search of peer-reviewed research papers published before June 2022 was conducted using the electronic databases CINAHL, Embase, Medline, PsycInfo, Social Science Database and Web of Science. This resulted in the screening of 7101 papers 27 of which matched the inclusion criteria. A quality appraisal was conducted using the Mixed Methods Appraisal Tool. Findings show that papers often lack a clear definition of resilience and application of resilience theory within the studies, although many of the papers conceptualised resilience as either a trait, process or an outcome. However, resilience was rarely the primary focus of the studies and was researched using a variety of measurement instruments and conceptual frameworks. Given the socioeconomic disparities, diverse social relations, histories of discrimination and stigma, and acts of resistance that have shaped the lives of older LGBT+ populations, resilience is a topic of growing interest for researchers and practitioners. Clear definitions of resilience and application of resilience theory could help improve methods used to study the concept and lead to more robust findings and the development of effective interventions. Greater clarity on the concept of resilience could also broaden the focus of research that informs policies and practice, and support practitioner training in resilience and the particular experiences of older LGBT+ adults.

## Introduction

Population ageing within global demography is one of the most significant social transformations of the twenty-first century. Projections show that by 2050, the proportion of people over 65 years will increase from 1 in 11 to 1 in 6 [1]. The diversity of the ageing population is a significant factor in understanding any challenges and opportunities in the way in which we respond to demographic changes [2]. While lesbian, gay, bisexual, transgender and gender diverse people (LGBT+) face similar challenges to their heterosexual and cisgender peers in later life, an established evidence base demonstrates specific health, social and structural inequalities for LGBT+ older people [3–5]. (We use the term LGBT with a plus (+) sign to signal inclusion of the wide diversity of sexual and gender identities, unless the paper being cited is focused on specific identities). Inequalities are compounded by the cumulative effects of lifelong exposure to prejudice, discrimination, criminalisation [6] and environmental factors nuanced by a wide range of intersecting identities, including socio-economic status, culture, race and ethnicity, disability and religion [7]. These unique circumstances impacting on ageing experience for LGBT+ people pose risks linked to minority stress [8] and stress adaptation in later life [9]. Minority stress posits that in comparison to the heterosexual and cisgender community the stigma and resulting discrimination experiencd by sexual and gender minority people creates a multitude of stressors that heighten their risk of negative physial and mental health outcomes. Some older LGBT+ people are more successful than others in adapting and coping with ageing: those with strong psychological and social resources are likely to enjoy better health and practice more health promotion behaviours [3, 10]. However, the design of effective interventions to promote such positive adaptations is not yet well understood. In addition, with the increased documented exposure of LGBT+ people to social and culturally embedded discrimination, there has been growing research interest in the role of resilience in promoting wellbeing in LGBT+ individuals, communities and populations.

A wider focus on resilience in later life has led to burgeoning research alongside debates about the ambiguities and methodological limitations of the research itself [11]. There is no universal definition of resilience [12] which is often described as a dynamic concept that may be researched as a trait, a process or an outcome [13]. Resilience has been defined as ’both the capacity of individuals to navigate their way to the psychological, social, cultural and physical resources that build and sustain their wellbeing, and their individual and collective capacity to negotiate for these resources to be provided and experienced in cultural meaningful ways’ [14, p. 17]. It is also considered something that can be taught or learnt, as an individual characteristic or trait, and as a coping process in response to one’s changing physical and social environment [11]. Resilience as a personal trait helps individuals cope, adjust and develop, inoculating them against the impact of adversity or traumatic events [13, 15]. Resilience can be regarded as a function or behavioural outcome that can conquer and help individuals to recover from adversity [16, 17]. It can be a process in which individuals actively adapt to and recover from major adversities [18, 19]. In older adult research, resilience is often described or examined through a life course lens where the potential to adapt to the challenges, changes and disruption to adversity associated with normative ageing [20, 21] can lead to the use of positive coping practices [22]. Allen et al’s [11] definition of resilience speaks to the processes of being mindful of and prioritising behaviours, thoughts, and feelings that facilitate contentment within one’s specific developmental, physical, emotional, and spiritual context. Angevaare et al’s [23] concept review in ageing research identified three common features of descriptions of resilience: a stressor, a response and a mechanism, all of which are dynamic and emphasise the importance of the context in achieving resilience.

More critical commentators [24, 25] take a theoretical and political critique of how the concept of resilience has been applied in the social science literature and the implications of the resilience discourse. They argue that trait, process and outcome perspectives are focusing on our ‘subjectivity’ and as a consequence discussions about the ‘outside world’ [25, p. 40] and the radical transformation needed to challenge established social ‘systems’ [24, p. 254] are closed off or subjugated. In other words the emphasises on ’individual responsibility’ [24] is a form of neoliberal governance that places onus on individuals and communities as consumers to become resilient and adaptable to external stressors, and in so doing the inequalities and oppressive social structures which create the need for resilience in the first place go unquestioned and depoliticised.

Looking specifically at LGBTQ (authors included Queer identities) health research, Colpitts and Gahagan [26] found that although the concept of resilience emerged as a key conceptual framework to advance a strengths-based approach and suggested ways in which resilience is defined and measured in relation to LGBTQ populations, it remains a contested concept. De Lira and de Moreis in their review of the LGB literature [27] note that in the light of the simultaneous interaction between the individual, family, and social contexts, and their contributions to the process of resilience the conceptual dimensions of resilience must be further integrated to provide a more accurate description of its relational and systemic nature.

In relation to LGBT+ ageing, some of the research conducted with populations experiencing physical or mental health difficulties has identified different factors and processes, which form pathways to resilience [28, 29]. It is also important to take account of the intersectionality of social, cultural, economic and other factors that shape resilience in later life [29]. Higgins et al. [30] caution that while negative experiences can adversely affect LGBT+ peoples mental health and emotional wellbeing, this perspective may also unwittingly lead to LGBT+ identities being viewed as pathological. Herek et al’s [31] social psychological framework for understanding stigma in sexual minority adults articulates how individual personal acceptance of sexual stigma as a part of ones own value system is internalised by adapting one’s self-concept to be congruent with the stigmatizing responses of society. This has the potential to obscure and silences any potential for the development of unique strengths and skills that can be characterised as resilience [32] which in turn go unrecognised or undervalued. As a rapidly growing field of enquiry, knowing more about the degree to which theories and the nature of theories can shape the integration of knowledge will promote understanding and support for LGBT+ lives [33, 34]. How resilience is conceptualised and defined in research could help improve definition and methods used to study it and help deepen our understanding about what interventions might be effective in promoting resilience and quality of later life [29].

## Aims of the review

The aim of this systematic review was to examine how resilience is defined in LGBT+ ageing research and how it is studied.

## Methodology

Given the aims and objectives of the review, we conducted a systematic mixed study review as described by Pluye and Hong [35]. Mixed study reviews have the advantage of allowing for a more complete analysis of the available evidence drawing from qualitative, quantitative and mixed methods studies [36]. The complexity and lack of consensus within the phenomenon of interest informed the need for integrating qualitative and quantitative papers and this emerged when articulating the research question [37]. A data based convergent synthesis design was adhered to [35, 37] and we present the findings of the systematic searches using PRISMA guidelines [38].

### Search strategy and study selection

A comprehensive and systematic search strategy was constructed by adapting strategies in previous studies coupled with input from the project team. This was trialled in a sample database, edited based on comments from the team, and implemented by the information specialist [GS]. The search string explored the three main concepts of ‘LGBT+’ [e.g. Lesbian; Gay; Bisexual; Transgender], ‘older’ and ‘resilience’ using synonyms, controlled vocabulary and Boolean operators as appropriate (see S1 Appendix. Example of search terms used in Medline (Ebsco)). CINAHL (Ebsco), Embase (Elsevier), Medline (Ebsco), PsycInfo (Ebsco), Social Science Database (ProQuest), and Web of Science (Clarivate) were all searched from inception until 17 June 2022. The protocol for conducting this review was registered with PROSPERO (registration number: CRD42021249093).

### Eligibility criteria

Given the aims of the review, the focus was on peer-reviewed papers in English only. Studies which reported primary research with older LGBT+ individuals who were aged 50 and above were included. Depending on the type of review people at the outset may define their concept or phenomenon of interest very tightly and use that definition as an inclusion criterion. However, as our review was focused on how researchers conceptualised and defined resilience within their research, we did not have a strict definition of resilience, although our reading and knowledge of the concept did inform our thinking on the search terms used. Studies included could be qualitative, quantitative or mixed methods as long as there was a clear mention of resilience within the paper. Resilience could be a primary or secondary focus of the research study or have emerged from participant’s narratives in qualitative studies. Systematic reviews, case studies, randomised controlled trials, intervention studies and descriptive/discussion papers were excluded (see S1 Table. Inclusion/Exclusion criteria).

### Screening and selection

**Fig 1:**
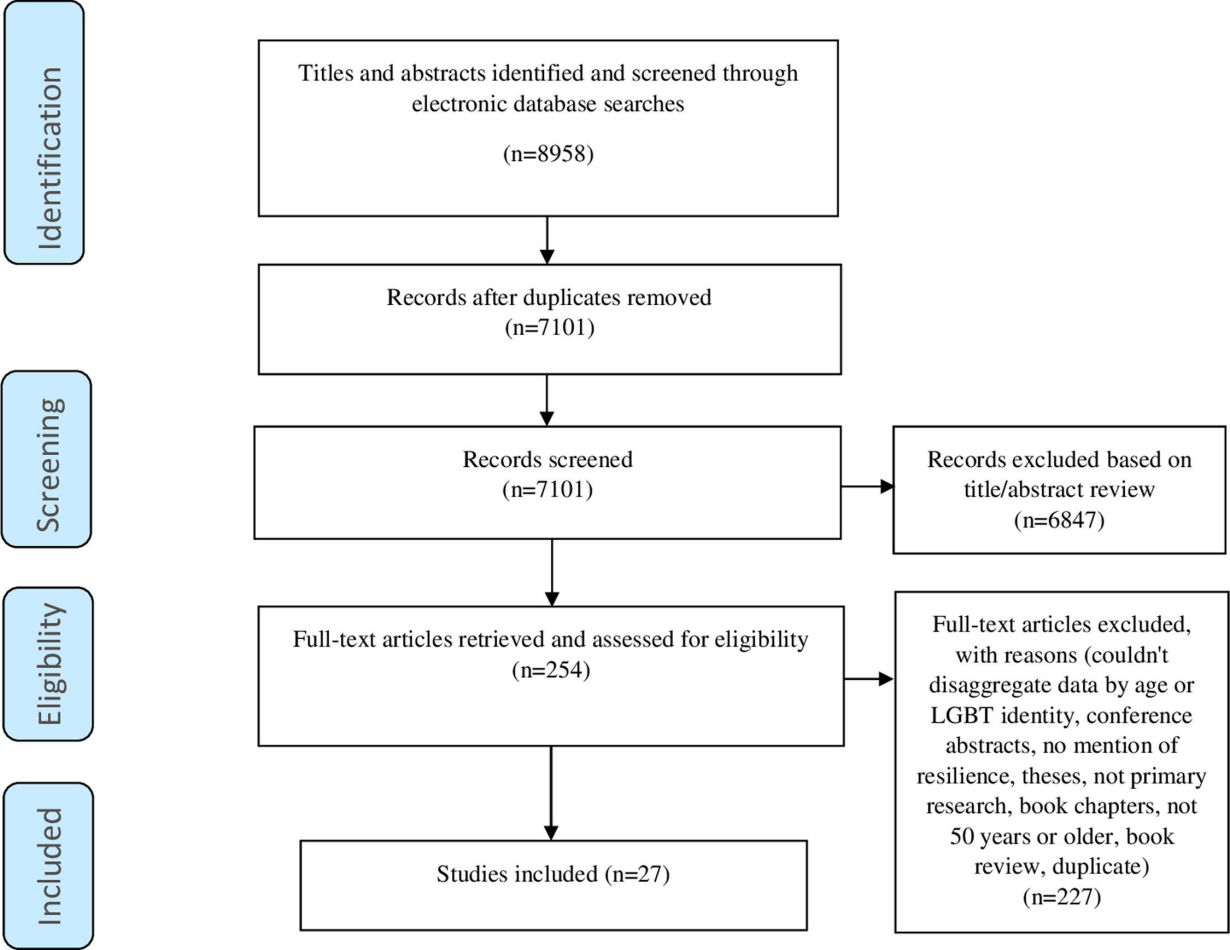
PRISMA flow diagram of selection process.

Following the database searches, all citations were uploaded into Covidence screening software (https://www.covidence.org) and any duplicates removed. Each title and abstract were independently assessed against the inclusion and exclusion criteria for eligibility by two members of the review team. Where there was difference in assessment, a third member of the team reviewed the title and abstract and made the final decision to include or exclude. Out of the 7101 papers reviewed at title and abstract level, 254 were put forward for full text review. These papers where once again assessed against the inclusion and exclusion criteria for eligibility by two members of the review team. Out of the 254 citations in total 27 papers were eligible for inclusion. Fig 1 represents the PRISMA diagram.

### Quality assessment

Quality appraisal in a mixed study systematic review has been described as complex [39] and different approaches have been used [40]. We used the Mixed Method Appraisal Tool or MMAT [41], specifically designed for quality appraisal in mixed study reviews. The MMAT includes two general screening questions and a further five methodological quality criteria for common types of methodologies which permits the critical appraisal of mixed study reviews within a single tool and specific quality criteria for mixed methods studies [42]. An affirmative response to both the initial screening questions (are there clear research questions and do the collected data allow to address the research questions) indicate that the paper meets initial criteria for screening. As per the MMAT user instructions, two of the authors independently assessed the quality of the papers and met to discuss and reach consensus about the quality of the individual papers. Excluding papers that are of low methodological quality is discouraged by the authors of the MMAT.

All papers (n = 27) met the baseline for empirical studies following initial screening. Of the 27 papers included there were 13 qualitative and 14 quantitative papers. Collectively the quality of the papers ranged from average to good using the MMAT. All quantitative papers were appraised as cross-sectional descriptive studies based on their methodology. For the quantitative studies, only one study met all the quality indicators [43] with the others failing to meet at least one of the MMAT criteria. For the most part, studies were not representative of the target population or used non-probability convenient sampling. In addition, it was not clear in eight of the quantitative papers if the risk of non-response bias had been addressed [44–51]. Only four qualitative studies met the full criteria following initial screening [52–55]. All papers were subsequently included in the extraction and analysis phase as advocated by Hong et al. [42].

### Data extraction and analysis

Two authors designed and piloted the data extraction template, which included the aim of the research, sample characteristics, and details on the study methodology. Any data on resilience, particularly how resilience was defined and conceptualised, if resilience was the primary or secondary focus (objective) of the study, and whether it was an entry or an outcome of the study was extracted. For the purpose of this review, studies that had resilience as a stated or inferred objective were classified as entry point studies and those where resilience was a topic inductively emerging from the data were classified as outcome studies. Details on the tools to measure resilience and conceptual frameworks used, were also extracted (see Table 1). In the qualitative papers, data findings relating to the review aims were extracted verbatim, whereas in the quantitative papers, data was transformed (‘qualitised’) in line with the convergent synthesis design that was adopted [37]. Data was extracted manually from each paper by two authors who then met to discuss the extraction and finalise the included data. Once the templates were populated with data, meetings were held with all authors who tabulated and summarised the data given the aims of this review, using a descriptive approach. This process included establishing an audit trail which articulated the process from paper selection through to data extraction, tabulation and data visualization which was checked by an author who acted as moderator who critiqued the process. In addition, regular meetings with all team members ensured that the team were clear on issues such as inclusion criteria, data selection, analysis and presentation of findings.

**Table 1:** Overview of included papers.

**Qualitative studies**				
**Author (Date), Country**	**Study Design, Data Collection Methods/Analysis**	**Sample (size and characteristics)**	**Focus of the study (entry or outcome)** ^**1**^	**Definition of resilience and literature/framework utilisation**
Dziengel (2012) [52], USA	**Study design:** Secondary analysis of a subset of qualitative data. **Original study—data collection method:** Online survey with 6 open-ended questions about supports and threats to longevity of the couple relationship, which were the focus of the secondary analysis. **Data analysis for the secondary analysis:** Constructivist grounded theory methods.	**Sample size:** N = 156 (70 couples and 16 individuals whose partners didn’t complete survey) **Sample Characteristics:** **Gender identity:** Female (53.1%), Male (46.9%), other descriptors on gender identity not reported **Sexual orientation:** Gay (53.1%), Lesbian (39.3%), other not reported **Age:** 44–81 years (mean: 58.2) **Ethnicity:** Caucasian* (96.6%)	Entry ✓ Outcome **Commentary on focus**: Resilience was the **primary** focus of the secondary analysis of the subset data. **Objective:** To explore how participants describe resilience in their relationships.	a) Resilience conceptual framework b) Literature overview of resilience ✓ c) Resilience only mentioned Using interpretations of other writers, resilience is described as ‘a flexible equilibrium in that people are able to use additional supports even though they may never gain resolution of the loss and that resiliency is something that must be seen as a process and movement over time’ [52, p. 76].
Rowan & Butler (2014) [56], USA	**Study design:** Phenomenological qualitative approach and utilization of gerontology perspective framework. **Data collection method:** In-depth interviews on understanding the core meanings and the essence of experiencing alcoholism with specific focus on ‘(a) life history, (b) details of experiences, and (c) reflection of the meaning given to life events and circumstances’ [56, p. 181]. **Data analysis:** Phenomenological methods.	**Sample size:** N = 20 **Sample Characteristics:** **Gender identity:** Not reported **Sexual orientation:** Reported as lesbian **Age:** 50–70 (mean: 57.6) **Ethnicity:** White (95%), African American (5%)	Entry Outcome ✓ **Commentary on focus**: Resilience is neither a primary or a secondary focus of the study but an **outcome** of the research and is included by the authors in the discussion and is briefly defined.	Resiliency emerges as an outcome of the study in relation to how participants saw themselves as resilient in handling life challenges. It is conceptualized as participants’ strengths and capacities to handle life challenges, contributing to their overall well-being. Based on this interpretation, resiliency is discussed in the context of literature that defines resilience as a way of dealing with life difficulties and ability to bounce back from adversity.
Foster et al. (2015) [57], USA	**Study design:** Exploratory retrospective qualitative study. **Data collection method:** Interviews with an overarching open research question on the processes and key factors in participants’ identity integration of being both gay/lesbian and Christian and a number of probes. No specific question about resilience was posed. **Data analysis:** Grounded theory methods.	**Sample size:** N = 27 **Sample Characteristics:** **Gender identity** was not used as a category. The sample was described as: Male (37%), Female (63%), other descriptors on gender identity not reported **Sexual orientation:** Reported as lesbian and gay **Age:** 25–80 (mean: 52.5) **Ethnicity:** White/Caucasian*/European American/Anglo Saxon (92.6%), Native American (3.7%), Human being (3.7%)	Entry ✓ Outcome **Commentary on focus**: The primary focus of the research is integration of orientation and faith. Despite the authors highlighting the resilience strategies LG Christians utilised in coming out and living out their sexual orientation within the literature, and constructing a theoretical model for LG Christian Spiritual Resilience, resilience appears to be a **secondary** focus of the study. **Objective**: To identify and understand the processes of integrating orientation and faith.	a) Resilience conceptual framework b) Literature overview of resilience ✓ c) Resilience only mentioned Drawing on a variety of other peoples’ work resilience is viewed as the ability to competently function in the face of life stressors, recover from life disruptions and meet future challenges. It is described as a unique individualised process of moving from ‘risk to psychological and spiritual healing’ using ‘behavioural and meaning-making strategies’ to develop protective factors [57, p. 192].
Elder (2016) [58], USA	**Study design:** Descriptive research. **Data collection method:** Semi-structured, qualitative interviews on the subjective experiences of participants in psychotherapy. Questions allowed participants to identify and share aspects of their therapy experiences and lives they considered important. **Data analysis:** Inductive thematic analysis.	**Sample size:** N = 10 **Sample Characteristics:** Gender (self-identify as)^*^: Female (40%), Male to female and back (10%), Male (30%), Trans man (10%), Transgender woman (10%), other descriptors on gender identity not reported **Sexual orientation:** Asexual (10%), Attracted to men (10%), Bisexual (20%), Gay men (10%), Heterosexual (20%), Lesbian (2), Straight (10%), other descriptors not reported **Age:** 60–83 **Ethnicity:** Black/African American (10%), Chinese (10%), White/Caucasian* (80%)	Entry Outcome ✓ **Commentary on focus**: Resilience is neither a primary or a secondary focus of the study but an **outcome** of the research and is included by the authors in the discussion.	Resilience emerges as an outcome of the study as a theme in relation to how participants demonstrated resilience against discrimination and oppression, which was interpreted as activism and is briefly addressed in discussion.
Higgins et al. (2016) [59], Republic of Ireland	**Study design:** Descriptive exploratory. **Data collection method:** In-depth face to face semi-structured interviews. **Data analysis:** Inductive thematic analysis.	**Sample size:** N = 36 **Sample Characteristics:** **Gender identity:** Male (61.1%), Female (30.5%), Transgender (5.6%), Other (2.8%), other descriptors on gender identity not reported **Sexual orientation:** Gay (61.1%), Lesbian (36.1%), Bisexual (2.8%) **Age:** 55–59 (61%), 60–64 (25%), 65–69 (8%), 70–74 (6%); (mean: 60.3) **Ethnicity:** Not reported	Entry ✓ Outcome **Commentary on focus**: Resilience was the **primary** focus of the study. **Objective:** To explore resilience processes among older LGBT adults.	a) Resilience conceptual framework b) Literature overview of resilience ✓ c) Resilience only mentioned Interpreting other writers work, the authors conceptualized resilience as a dynamic concept comprised of behavioural and meaning making attributes that can fluctuate through the life course. Resilience is understood as both individual and collective capacity of navigating psychological, social, cultural and physical resources that build and sustain well-being.
Boggs et al. (2017) [60], USA	**Study design:** Mixed qualitative descriptive design. **Data collection method:** Mixed methods (intercept interviews, focus groups, in-depth interviews and a town hall meeting). **Data analysis:** Audiotapes (focus groups and final interviews), notes and flipcharts (town hall and intercept interviews) were coded for key themes.	**Sample size:** N = 73 (intercept surveys (17), focus groups (14), town hall meeting (30), final interview (12)) **Sample Characteristics** (Focus Groups (n = 14)): Participants identified by facilitators as gay males in their 70s and lesbian females in their 60s **Ethnicity:** not reported **Sample Characteristics** (Surveys and Final Interviews (n = 29)): Gender identity and sexual orientation reported as ‘self-identify as’*: Female (69%), Lesbian (55%), Male (31%), Gay (31%), Transgender (7%), Bisexual (7%), Pansexual (3.5%), Queer (3.5%), Straight (3.5%), other descriptors not provided **Age:** 40–49 (3.5%), 50–59 (41.3%), 60–69 (45%), 70–79 (10.2%) **Ethnicity:** Not fully reported	Entry Outcome ✓ **Commentary on focus**: Resilience is neither a primary or a secondary focus of the study but an **outcome** of the research and is included by the authors in the discussion.	Resilience emerges briefly in participants’ narrations in relation to having coping skills to get through life and live to an old age. Authors briefly mention resiliency and resilience as part of strengths in discussion.
Handlovsky et al. (2018) [53], Canada	**Study design:** Grounded theory. **Data collection method:** In-depth interviews, loosely structured by a topic guide comprising several questions and probes. **Data analysis:** Grounded theory methods.	**Sample size:** N = 25 **Sample Characteristics:** **Gender identity** was not used as a category. The sample was described as: Men, other descriptors on gender identity not reported **Sexual orientation:** Reported as gay men **Age:** 40–76 (mean: 54) **Ethnicity:** Caucasian* (92%)	Entry ✓ Outcome **Commentary on focus**: Resilience was the **primary** focus. **Objective:** To explore how middle-aged and older gay men developed resilience over the life course to promote health and wellness.	a) Resilience conceptual framework b) Literature overview of resilience ✓ c) Resilience only mentioned Referencing other researchers work, resilience is understood as an individual process of ‘adaptation’ when facing ‘adversity’ that help battle ‘negative effects’ of peoples’ exposure to risks. Risks and internal and external protective processes are both integral to developing resilience [53, p. 1474].
Green & Wheeler (2019) [61], USA	**Study design:** Narrative research. **Data collection method:** Semi-structured interviews. **Data analysis:** Theoretical thematic analysis informed by a Health-Care Utilization theoretical framework.	**Sample size:** N = 10 **Sample Characteristics:** **Gender identity:** Cisgender men **Sexual orientation:** Gay/homosexual men **Age:** Mean: 58.3 **Ethnicity:** African American/Black (60%), Caucasian*/White (40%)	Entry Outcome ✓ **Commentary on focus**: Resilience is neither a primary or a secondary focus of the study but an **outcome** of the research and is included by the authors in the discussion.	Resilience emerged in participants’ narratives in the context of how resilience buffered and lessened negative experiences in health care. Sense of resilience and the process of developing it is recognised in discussion by the authors and although not clearly defined it is discussed as ‘the ability to recover and grow from negative experiences’ and as ‘having something within yourself, some inner faith to fall back on’ [61, p. 4].
Jen & Jones (2019) [54], USA & UK	**Study design:** Cross national secondary analysis of two independently conducted studies. **Original studies—data collection methods:** In depth narrative interviews (Study 1), in-person interviews (Study 2). **Data analysis:** Thematic analysis (original Study 1), Foucaldian discourse analytic approach (original Study 2). Secondary analysis of qualitative data compared themes and discursive interpretations across the two completed studies.	**Sample size:** Study 1 (UK): N = 12, Study 2 (USA): N = 12 **Sample Characteristics Study 1:** **Gender identity:** Female (50%, 25% with trans histories), Male (33%), Pangendered (8.5%), Queer femme and trans (8.5%) **Sexual orientation:** Bisexual (50%), Other preferred identities including lesbian, gay, queer, pansexual, with bisexual histories. **Age:** 51–83 (mean: 64) **Ethnicity:** White (100%) **Sample Characteristics Study 2:** **Gender identity:** Cisgender women (100%) **Sexual orientation:** All identified as bisexual but also used other identities such as queer (58%), ‘hasbian’ (17%), lesbian (17%), pansexual (8%) **Age:** 60–77 (mean: 65) **Ethnicity:** White/Caucasian* (75%), Black/African American (17%), Asian American (8%)	Entry Outcome ✓ **Commentary on focus**: Resilience is neither a primary or a secondary focus of the study but an **outcome** of the research and is included by the authors in the discussion.	Resilience emerged in participants’ narratives as part of a theme on HIV/AIDS epidemic and participants in the study mentioned resilient communities’ efforts around HIV/AIDS. Authors include resiliency and resilience in the discussion and relate them to broader cultural and historical events and intersectional identities recognised in the narratives.
Nelson-Becker & Thomas (2020) [62], UK, USA & Canada	**Study design:** Secondary analysis of qualitative data from two studies. **Original study data collection method:** Narrative dyad interviews with couples on motivations to marry, wedding and civil partnership ceremonies (Study 1). Interviews focusing on life challenges and spiritual and religious understandings among older Black and Jewish persons (Study 2). **Data analysis of original study:** Theoretical framework (Study 1), Grounded theory (Study 2). **Secondary data analysis:** Informed by Resilience properties in Aging Framework and the use of selective coding.	**Sample size:** Study 1: N = 55 (LGBQ spouses and couples); Study 2: N = 75 (older Black and Jewish persons) **Sample Characteristics:** **Gender identity:** Not reported in either study **Sexual orientation:** Not reported in either study (in study 1 the couples/spouses are reported as being LGBQ) **Age:** Study 1, statements are from participants in their 50’s and older; Study 2, Jewish and Black group (median: 81 and 75, age range 58–92) **Ethnicity:** Not reported in study 1, only reported as Jewish and black in study 2	Entry ✓ Outcome **Commentary on focus**: Specifically spiritual resilience was the **primary** focus of the study. **Objective:** To describe how older persons respond to life challenges and spiritual struggles through spiritually resilient responses.	a) Resilience conceptual framework ✓ b) Literature overview of resilience ✓ c) Resilience only mentioned A resilience in Aging Framework [63] is used with the addition of the concept transilience, which was developed by Canda et al. [64] and ‘addresses the capacity to transcend ego and body-bound limits as we encounter new experiences’ [62, p. 3]. A thorough resilience literature review by the authors conceptualizes resilience as something that brings balance to older people and is understood as a capacity that helps manage difficulties and stress and overcoming barriers. Process, outcome and trait aspects of resilience are all recognized.
Bower et al. (2021) [65], USA	**Study design:** Narrative methodology. **Data collection method:** Face-to-face and video conference interviews in which participants were asked to share artefacts they felt enriched their narrative. **Data analysis:** Narrative Analysis, with the coding process guided by generativity theory, critical feminist perspective and queer theory.	**Sample size:** N = 18 **Sample Characteristics:** Gender^*^: Cisgender male (66%), Cisgender female (22%), Transgender (12%) Sexuality*: Gay (72%), Lesbian (22%), Straight (6%) **Age:** 46–76 years **Ethnicity:** White, Non-Hispanic (100%)	Entry ✓ Outcome **Commentary on focus**: Resilience was the **secondary** focus of the study, while the main foci were: multiple stigmas in relation to LGBTQ and aging and cultural generativity. **Objective:** To explore the way older LGBTQ+ adults find meaning in response to stigma and trauma.	a) Resilience conceptual framework b) Literature overview of resilience c) Resilience only mentioned ✓ Resilience not defined. However, it was mentioned in relation to other concepts that were the primary focus of the paper.
Haldane et al. (2021) [55], Canada	**Study design:** Descriptive research. **Data collection method:** Semi-structured interviews on participants’ descriptions of significant events, and experiences of hope related to the LGBTQ+ community. **Data analysis:** Constant comparative method using open and analytical coding.	**Sample size:** N = 7 **Sample Characteristics:** **Gender identity:** Not reported Sexuality*: Gay men (71%), Lesbian women (29%) **Age:** 50–70 years old **Ethnicity:** Not reported	Entry ✓ Outcome **Commentary on focus**: Resilience was the **primary** focus of the study as the study explored how prominent ‘sexual orientation minority (SOM) elders’ perceived the LGBTQ+ community as developing hope and resiliency in relation to major events of LGBTQ+ rights development. **Objective:** To explore ‘how did significant local and national historical events impact the SOM community at the time’ and ‘what did these events mean for fostering hope for the SOM community?’ [55, p. 5]	a) Resilience conceptual framework b) Literature overview of resilience ✓ c) Resilience only mentioned Drawing from a variety of other authors, resiliency in this paper is conceptualised as individuals’ ability to persist difficulties, making them less vulnerable to adversities, resulting in better than expected outcomes. Resiliency is understood to develop over time and includes an interplay of socially, historically, culturally and otherwise conditioned factors.
Stinchcombe et al. (2021) [66], Canada	**Study design:** Constructivist grounded theory. **Data collection method:** 10 semi-structured focus groups held in community settings. **Data analysis:** Constructivist grounded theory methods.	**Sample size:** N = 61 **Sample Characteristics:** **Gender identity:** Cis-Man (49%), Cis-Woman (36%), Transwoman (13%), Missing data (2%), other descriptors not reported **Sexual orientation:** Homosexual (75%), Heterosexual (2%), Bisexual (15%), Other (6%), Missing data (2%) **Age:** 58–79 (mean: 67) **Ethnicity:** Caucasian* (90%), South Asian (5%), Black (1.6%), Métis (1.6%), Inuit (1.6%)	Entry Outcome ✓ **Commentary on focus**: Resilience is neither a primary nor a secondary focus of the study but an **outcome** of the research.	Resilience emerged briefly in participants’ narratives in relation to how historical experiences led to development of resilience in old age.
**Quantitative studies**				
**Author (Date), Country**	**Data Collection Methods/Analysis**	**Sample (size and characteristics)**	**Focus of the study (entry or outcome)** ^**1**^	**Definition of resilience and literature/framework utilisation**
Fredriksen-Goldsen et al. (2013)^2^ [47], USA	**Study design:** Quantitative cross-sectional. **Data collection method:** Mail and electronic survey utilising multiple existing tools to measure health outcomes, health indicators, risk factors, protective factors and background characteristics. Resilience was not directly measured. **Data analysis:** Descriptive and inferential statistics were performed using STATA/IC for Windows (version 11.2).	**Sample size:** N = 2349 **Sample Characteristics:** Gender*: Women (35.3%), Men (64.7%) (Transgender participants were excluded from the sample) **Sexual orientation:** Lesbian (32.8%), Gay (61.9%), Bisexual men (2.8%), Bisexual women (2.5%) **Age:** Mean: 66.88 (age range data not reported) **Ethnicity:** Non Hispanic White (87.1%)	Entry ✓ Outcome **Commentary on focus**: Despite using a resilience conceptual framework [67], resilience itself was not directly measured as a construct and the main focus of the study was on a range of factors that influence mental and physical health. Resilience was researched in connection to these factors and was therefore a **secondary** focus of the study. **Objective**: To examine influence of key health indicators, risk and protective factors to health outcomes using a resilience framework.	a) Resilience conceptual framework ✓ b) Literature overview of resilience ✓ c) Resilience only mentioned A resilience conceptual framework [67] was used in which the association between five dimensions (health outcomes, health indicators, risk factors, protective factors and background characteristics) was examined. The framework reflects the understanding of resilience as a dynamic process in which risks and protective factors interplay. Resilience can be observed in individuals, families and communities, manifesting as competences, capacities and behavioural patterns that are beneficial when facing adversity.
Fredriksen-Goldsen et al. (2014)^2^ [68], USA	**Study design:** Quantitative cross-sectional. **Data collection method:** Mail and electronic survey utilising multiple existing tools to measure health outcomes, health indicators, risk factors, protective factors and background characteristics. Resilience was not directly measured. **Data analysis:** Descriptive and inferential statistics were performed using STATA/IC for Windows (version 11.2).	**Sample size:** Total sample size: N = 2546, Transgender data subset: N = 174 **Sample Characteristics** (transgender data subset): **Gender identity:** Men (37%), other descriptors on gender identity not reported **Sexual orientation:** Not reported **Age:** Mean: 60.97 (age range data not reported) **Ethnicity:** Non-Hispanic white (79.07%), Native American (6.98%), African American (4.65%), Hispanic (3.49%), Multiracial (2.33%), Asian/Pacific Islander (1.74%), Other (1.74%)	Entry ✓ Outcome **Commentary on focus**: Despite using a resilience conceptual framework [67], resilience itself was not directly measured and the main focus of the study was to ‘assess the effects of gender identity on physical and mental health outcomes and explore the mediating role of key health indicators, and risk/protective factors’ [68, p. 490]. Resilience was researched in connection to these factors and was therefore a **secondary** focus of the study. **Objective**: ‘To identify modifiable factors that account for health risks in the transgender older adult population’ [68, p. 488].	a) Resilience conceptual framework ✓ b) Literature overview of resilience c) Resilience only mentioned A resilience conceptual framework was used in which the association between health outcomes, health indicators, risk factors, protective factors and background characteristics were examined. Resilience itself was not defined.
Fredriksen-Goldsen et al. (2015)^2^ [69], USA	**Study design:** Quantitative descriptive, comparative and cross-sectional. **Data collection method:** Paper and online survey measuring outcome variables: physical and mental health, quality of life (QOL); and explanatory variables: social risks, identity management resources, social resources, health-promoting behaviours, socioeconomic resources and background characteristics. **Data analysis:** Descriptive statistics, bivariate analysis and multivariate linear regression was used.	**Sample size:** N = 2463 **Sample Characteristics:** Gender*: Female (36.34%), **Gender identity:** Transgender (4.12%), other descriptors on gender and gender identity not reported Sexual identity*: Gay men/Lesbian (92.94%), Bisexual (7.06%) **Age:** 50–64 (43.77%), 65–79 (46.2%), 80+ (10.03%) **Ethnicity:** White (86.85%), Other (5.47%), Hispanic (4.25%), African American (3.43%)	Entry ✓ Outcome **Commentary on focus**: Despite using The Resilience Framework, resilience itself is a **secondary** focus of the study as it was researched in relation to health-related QOL. Five dimensions related to QOL are examined in the paper. **Objective**: To investigate relationship between physical and mental health-related quality of life (QOL) and covariates by age group using a Resilience Framework.	a) Resilience conceptual framework ✓ b) Literature overview of resilience ✓ c) Resilience only mentioned A resilience framework was used in which the association of five dimensions (health outcomes, health indicators, risk factors, protective factors and background characteristics) as they relate to QOL, as an indicator of successful aging were examined. Resilience itself is understood as a set of resources and capacities that are utilised when facing adversity.
Fredriksen-Goldsen et al. (2016)^2^ [48], USA	**Study design:** Quantitative descriptive, comparative and cross-sectional. **Data collection method:** Mail and electronic survey. The Health Equity Promotion Model [70] was utilised and multiple existing tools and scales were used to measure: sexual identity factors, social and socioeconomic resources, mental and physical health and background characteristics. Resilience was not directly measured. **Data analysis:** Bivariate correlations and structural equation modelling (SEM) was conducted to test the hypothesised model.	**Sample size:** Total sample size: N = 2463, Bisexual data subset: N = 174 **Sample Characteristics** (bisexual data subset): Gender*: Male (47.7%), Female (46.55%), Other (5.75%), other descriptors on gender not reported **Sexual orientation:** Bisexual (100%) **Age:** 50–95 (mean: 65.69) **Ethnicity:** Non-Hispanic White (86.13%), Person of color (13.87%)	Entry ✓ Outcome **Commentary on focus**: The focus of the study was utilising The Health Equity Model [70] of which resilience is a part off. Resilience is therefore a **secondary** focus of the study, while the main focus is investigating direct and indirect associations between sexual identity and health via sexual identity factors, social resources, and socioeconomic status. **Objective**: To investigate hypothesised mechanisms accounting for health disparities between bisexual older adults and lesbian and gay older adults and reveal potentially protective pathways.	a) Resilience conceptual framework ✓ b) Literature overview of resilience c) Resilience only mentioned ✓ Resilience was not defined but was recognised as part of The Health Equity Promotion Model [70], which is an intersectional life course framework, used to identify multiple potential mechanisms that influence the aging process.
King & Richardson (2016) [71], USA	**Study design:** Quantitative descriptive, cross-sectional design. **Data collection method:** Online survey with 91 questions on health, health-promoting variables, psychosocial stress, and personal coping approaches. Multiple tools were used including The Wagnild and Young Resilience Scale to measure resilience [72]. **Data analysis:** Stepwise regression analysis was used.	**Sample size:** N = 316 **Sample Characteristics:** **Gender identity** was not used as a category. The sample was described as: Men other descriptors on gender identity not reported **Sexual orientation:** Reported as gay men **Age:** 46–64 (73%), 65+ (27%) **Ethnicity:** Caucasian* (94%), Latino (2.5%), African-American (1.6%), Asian (0.62%), Pacific Islander (0.32%), Native American (0.32%), bi- or multi-racial (0.32%), other (0.32%)	Entry ✓ Outcome **Commentary on focus**: Resilience was the **primary** focus of the study as it was considered alongside other predictors (stigma, discrimination, internalised homophobia) of mental distress in gay men. **Objective**: To test the hypotheses that mental distress is positively associated with stigma, discrimination, and internalised homophobia, but negatively related to resilience and those with more income and social support resources.	a) Resilience conceptual framework b) Literature overview of resilience ✓ c) Resilience only mentioned Drawing on other researchers work, authors conceptualized resilience as a dynamic process in which positive adaptation and effective coping strategies are maintained by individuals and help them cope with adversity in positive ways. Consequently this promotes health and well-being and acts as a buffer to physical and mental health distress.
Emlet et al. (2017)^3^ [43], USA	**Study design:** Quantitative descriptive, cross-sectional. **Data collection method:** The data came from a sub sample from Aging with Pride study. Five distinct domains were studied: background characteristics, HIV-related factors, adverse experiences, psychosocial characteristics, and outcome variables. Resilience was measured by the 3-item scale [73, 74]. **Data analysis:** Multivariate linear regression and hierarchical model were used.	**Sample size:** N = 335 **Sample Characteristics:** **Gender identity** was not used as a category. The sample was described as: Men, other descriptors on gender identity not reported **Sexual orientation:** Gay men (85.87%) (other data not reported) **Age:** 50–84 (mean: 58.32) **Ethnicity:** Non-Hispanic White (67.77%) (other data not reported)	Entry ✓ Outcome **Commentary on focus**: Resilience was the **primary** focus of the study as it aimed to examine both resilience and mastery as separate psychological resources that relate to each other but may be influenced by different factors in structural-environmental and individual contexts. **Objective**: ‘To examine HIV-related factors, adverse experiences, and psychosocial characteristics that are associated with resilience and mastery’ [43, p. S42].	a) Resilience conceptual framework b) Literature overview of resilience ✓ c) Resilience only mentioned Interpreting other researchers’ work on resilience, the authors understood resilience as consisting of different components (individual, interpersonal and environmental) that help individuals to adapt to risks and other age and non-normative identity related negative events. Main element resulting in resilience developed in a persons’ life is therefore adversity.
Fredriksen-Goldsen et al. (2017)^3^ [10], USA	**Study design:** Quantitative descriptive, cross-sectional. **Data collection method:** Paper and online surveys used existing scales measuring marginalization, psychological and social resources, mental and physical health, health behaviour and background characteristics. No specific measurements of resilience were conducted. **Data analysis:** Survey weights were applied, distributions of study variables were examined and bivariate correlations were computed between study variables. The hypotheses were tested using structural equation modelling.	**Sample size:** N = 2415 **Sample Characteristics:** Gender*: Female (43.17%), Male (50.76%), Other (16.6%) other descriptors on gender identity not reported **Sexual orientation:** Gay men or Lesbian (72.49%), Bisexual (17.19%), Other (10.32%), other descriptors not reported **Age:** 50–98 (mean: 61.45) **Ethnicity:** Non-Hispanic White (78.05%) (other data not reported)	Entry ✓ Outcome **Commentary on focus**: Resilience was a **secondary** focus of the study as the authors argue in the paper that a number of key variables will influence resilience. The main focus of the study was testing a structural equation model linking numerous dimensions (see column 2), for which Health Equity Promotion Model [70] was utilized. **Objective:** To investigate pathways by which LGBT older adults experience resilience, risk, and marginalisation and their relationship to attaining positive health outcomes.	a) Resilience conceptual framework ✓ b) Literature overview of resilience c) Resilience only mentioned ✓ Resilience was not defined but was recognised as part of The Health Equity Promotion Model, which is an intersectional life course framework, used to identify multiple potential mechanisms that influence the aging process.
Monin et al. (2017) [50], USA	**Study design:** Quantitative descriptive, comparative and cross-sectional. **Data collection method:** Survey data from the National Health and Resilience in Veterans Study (NHRVS).The study used a variety of scales to assess mental and physical health status, social support and exposure to trauma. No specific resilience scale was used. **Data analysis:** Multi-step statistical analyses, bivariate differences and adjusted logistic regression analyses.	**Sample size:** Total sample size: N = 3095, sexual minority data subset: N = 102 **Sample Characteristics** (sexual minority data subset): **Gender identity:** Male (76.5%), other descriptors on gender identity not reported **Sexual orientation:** Bisexual (50%), Gay (38.2%), Lesbian (11.8%) **Age:** Sexual minority sample: 23–89 (mean: 55.5) **Ethnicity:** Caucasian* (75%)	Entry ✓ Outcome **Commentary on focus**: Resilience was a **secondary** focus of the study which sought to examine whether sexual minority status confers vulnerability or resiliency in older adults, thus mental health was the primary focus of the study. **Objective:** To identify the mental health needs of older and younger sexual minority and heterosexual US veterans and examine whether sexual minority status confers vulnerability or resiliency in older adulthood.	a) Resilience conceptual framework b) Literature overview of resilience c) Resilience only mentioned ✓ Resilience not defined and some minor references to resilience in literature were made.
Cortes et al. (2019) [45], USA	**Study design:** Quantitative descriptive, comparative and cross-sectional. **Data collection method:** Online survey of LGBT identified veterans in which resilience was not directly measured but included measurements of demographics, depression, anxiety, use of alcohol, LGBT identity, negative identity, perceived heterosexist harassment, rejection, discrimination and outness. **Data analysis:** T-tests and multivariate logistic regression were used.	**Sample size:** N = 254 (older than 50 years (128), younger than 50 years (126)) **Sample Characteristics:** **Gender identity and sexual orientation** were not used as categories but reported under ‘LGBT identity’: Gay Man (31.9%), Lesbian Woman (21.7%), Bisexual (6.7%), Transwoman (31.1%), Transman (8.7%); over 50: 39.1%, 22.7%, 2.3%, 32%, 3.9% **Age:** 19–78 (mean: 47.3), over 50 (mean: 59.7) **Ethnicity:** White (78.2%), Hispanic (6.3%), Black/African American (5.2%), Multiple races (8.7%), Other (1.6%), Missing (0.8%), over 50: 81.9%, 5.5%, 3.1%, 7.1%, 2.4%, 0.8% respectively	Entry ✓ Outcome **Commentary on focus**: Resilience was a **secondary focus** of the study and was interpreted and researched in relation to anxiety, depression, alcohol use, harassment and outness. **Objective: ‘**To examine health and identity differences between older (50+) and younger (< 50) lesbian/gay women, gay men, bisexual, and transgender (LGBT) veterans’[45, p. 162], looking for evidence of older LGBT adults being more resilient than younger LGBT individuals.	a) Resilience conceptual framework b) Literature overview of resilience c) Resilience only mentioned ✓ Resilience not defined, but the authors allude to resilience as a focus when they reference evidence that ‘despite adversity’ LGBT older adults and older adults is general are more resilient due to tendency to focus on ‘positive stimuli’ and away from ‘negative stimuli’ [45, p. 163].
Batista & Pereira (2020) [44], Portugal	**Study design:** Quantitative descriptive, comparative and cross-sectional. **Data collection method:** Online survey including Connor-Davidson Resilience Scale 10 [15) and measurements of self-esteem, psychosympotmatology and sociodemographic information. **Data analysis:** Descriptive data analysis, Cronbach’s alpha and Pearson’s correlation tests were conducted.	**Sample size:** N = 201 **Sample Characteristics:** **Gender identity:** Men, other descriptors on gender identity not reported **Sexual orientation:** Gay men (80.6%), Bisexual (13.9%), missing data (5.5%) **Age:** 59–79 (mean: 58.85) **Ethnicity:** Not reported	Entry ✓ Outcome **Commentary on focus**: Resilience is one of the **primary** focuses although a literature review of resilience is not provided, but only mentioned. **Objective**: ‘To assess mental health disparities among gay and bisexual men over 50 years old, based upon their HIV status’ [44, p. 528]. The mental health indicators utilised sought to determine the respective predictive effects of resilience and self-esteem on the mental health of older gay and bisexual men.	a) Resilience conceptual framework b) Literature overview of resilience c) Resilience only mentioned ✓ Resilience is not defined, but the researchers recognize that there may be ‘adaptive factors’ that have been observed in relation to the discussed topics (minority stress, HIV etc.) in the paper, factors such as resilience that may ‘help mediate the impact of stigma and discrimination’ [44 p. 527].
Emlet et al. (2020)^3^ [75], USA	**Study design:** Quantitative descriptive, comparative and cross-sectional. **Data collection method:** Survey including various tools to measure health status, health outcomes, depressive symptomatology, LGBT-related discrimination and victimisation, LGBT-related micro-aggression, LGBT identity affirmation and stigma. To assess perceived resilience, a 3-item scale was utilised [73]. **Data analysis:** Distributions of background characteristics, health risk and promoting factors, and health outcomes by HIV status were examined, linear or logistic regressions were conducted, as were regression models and further statistical testing.	**Sample size:** N = 1344. Those living with HIV: N = 371, Those living without HIV: N = 973 **Sample Characteristics:** **Gender identity:** Men, other descriptors on gender identity not reported **Sexual orientation:** Gay (86.47% of those living with HIV and 81.15% of those living without HIV) (other data not reported) **Age:** Mean: 58.23 (HIV), 63.38 (non-HIV) (age range not reported) **Ethnicity:** Non-Hispanic White (59.64% living with HIV and 87.31% living withouth HIV)	Entry ✓ Outcome **Commentary on focus**: Resilience was a **secondary** focus of the study as part of conceptual framework of the Health Equity and Promotion Model [70]. **Objective**: To examine whether disparities exist in poor health and depressive symptomatology among older GB men (50+) with and without HIV, and if so, what risk/promoting factors account for those disparities.	a) Resilience conceptual framework ✓ b) Literature overview of resilience c) Resilience only mentioned ✓ Resilience was not defined but was recognised as part of The Health Equity Promotion Model [70], which is an intersectional life course framework.
Fleishman et al. (2020) [46], USA	**Study design:** Quantitative descriptive, cross-sectional. **Data collection method:** Online survey with 74 survey questions using 5-point Likert on sexual satisfaction, internalised homophobia, resilience, relationship satisfaction, sexual communication and demographic information. A 25-item Resilience Scale by Wagnild and Young [72] was used. **Data analysis:** Bivariate correlations and stepwise multiple regressions were conducted.	**Sample size:** N = 265 **Sample Characteristics:** **Gender identity:** Female (47%), Male (40.3%), Transgender (0.4), Other (0.4), Missing data (11.9%), other descriptors not reported **Sexual orientation:** Not reported, sample consisted of individuals in same-sex relationships **Age:** 60–64 (51.7%), 65–69 (32.8%), 70–75 (15.5%) **Ethnicity:** White/European American (81.9%), Multiracial (3.4%), Latino/ Hispanic/Latin American (1.9%), African/African American/Black/Carribean (1.5%), Native American/American Indian (0,4%), Asian/Asian American/South Asian/Pacific Islander/Hawaiian (0,4%), Missing data (10.5%)	Entry ✓ Outcome **Commentary on focus**: Resilience was one of the **primary** focuses of the study as it looked at the associations between internalised homophobia, resilience, sexual communication, relationship satisfaction and sexual satisfaction. **Objective:** To identify the associations between internalised homophobia, resilience, sexual communication, relationship satisfaction, and sexual satisfaction.	a) Resilience conceptual framework b) Literature overview of resilience ✓ c) Resilience only mentioned Uses the definition of resilience from the American Psychological Association as ‘the process of adapting well in the face of adversity, trauma, tragedy, threats, or significant sources of stress’ [46, p. 1980].
Pereira & Silva (2021) [51], Portugal	**Study design:** Quantitative descriptive, cross-sectional. **Data collection method:** Online survey comprising of sociodemographic information, scales on perceived social support, positive identity measure, resilience [15], successful aging and physical and mental health. **Data analysis:** Levels of association among various variables were assessed. Three multiple linear regressions were performed to determine the predictive effect of social support, positive identity, and resilience on successful aging perceptions and physical and mental health.	**Sample size:** N = 210 **Sample Characteristics:** **Gender identity:** Men, other descriptors on gender identity not reported **Sexual orientation:** Gay (85.3%), Bisexual (14.7%) **Age:** 50–80 (mean: 60.03) **Ethnicity:** Not reported	Entry ✓ Outcome **Commentary on focus**: Resilience was a **primary** focus of the study as it was recognised as one of the fundamental elements in need of researching in order to construct effective measures to support positive aging in older sexual minority men. **Objective:** To assess the levels of social support, positive identity, and resilience, and their relationship with successful aging among Older Sexual Minority Men.	a) Resilience conceptual framework b) Literature overview of resilience c) Resilience only mentioned ✓ Resilience is not clearly defined but recognised as a factor in successful aging and as an outcome of negative experiences by individuals. Authors reiterate other researchers in that the concept is hard to define and can be understood as an individuals’ trait or the influence of traits and coping experiences, furthermore it protects mental health and is related to processes of sexual minority individuals accepting a positive identity.
Lyons et al. (2022) [49], Australia	**Study design:** Quantitative descriptive cross-sectional design. **Data collection method:** A Survey including measurements of ageism, acceptance concerns, psychological distress, mental well being and resilience [74]. **Data analysis:** Descriptive statistics for all variables, followed by a correlation matrix of the key study variables.	**Sample size:** N = 613 **Sample Characteristics:** **Gender identity:** Cisgender men (70.5%), Cisgender women (29.5%) (TGNC participants were excluded) **Sexual orientation:** Gay men (70.5%), Lesbian women (29.5%) **Age:** 60–85 (mean: 65.83) **Ethnicity:** Not reported	Entry ✓ Outcome **Commentary on focus:** Resilience was a **secondary** focus of the study as it was examined as one of several mental health and well-being indicators. **Objective:** ‘To identify the extent to which experiences of ageism and sexuality acceptance concerns each predicted aspects of mental health and well-being and whether interactions between these two forms of stigma predicted poorer outcomes than either form alone’ [49, p. 2].	a) Resilience conceptual framework b) Literature overview of resilience c) Resilience only mentioned ✓ Resilience is not defined but merely mentioned as one of the mental health and well-being indicators examined in the study.

* Denotes the language used in the studies to specify demographics (e.g. gender, sexual identity or sexuality).

^1^ Studies with a stated or inferred objective were classified as entry point studies. Studies in which resilience inductively emerged from the data were classified as outcome studies.

^2^ Studies drawing from the same data set (Caring and Aging With Pride–data collected in 2010).

^3^ Studies drawing from the same data set (Aging With Pride–data collected in 2014).

## Findings

### Overview of studies

The 27 papers included represented 22 studies as four papers came from the ‘Caring and Aging with Pride’ study [47, 48, 68, 69] and three papers further analysed data from the ‘Aging with Pride’ study sample [10, 43, 75], which collected data in 2010 and 2014. The majority of the papers came from the USA (n = 17), three from Canada, two from Portugal, one from the Republic of Ireland and one from Australia. Three papers involved more than one country: USA/Canada (n = 1), USA/UK (n = 1) and USA/UK/Canada (n = 1). The papers were almost equally divided between quantitative (n = 14) and qualitative (n = 13) study designs. Quantitative studies focused on measuring variables that the researchers of the included papers considered represented resilience and the designs generally used descriptive, comparative and cross-sectional designs (n = 6), descriptive and cross-sectional designs (n = 6) and cross-sectional designs (n = 2). The emphasis of qualitative studies was on exploring participants’ perspectives or narratives on a variety of issues using qualitative descriptive (n = 4), grounded theory (n = 3), narrative methodology (n = 2), phenomenological (n = 1) approaches and secondary analysis (n = 3) of previously collected data. Data were collected mostly using one approach, namely individual interviews (n = 10), although in Bower et al’s [65] study participants were also asked to share artefacts they felt enriched their narrative interview. Other methods used were focus groups (n = 1), online survey with open-ended questions (n = 1) and one mixed methods study using interviews, focus groups, and a town hall meeting (n = 1).

Data analysis in four studies were informed by Charmaz’s [76] grounded theory approach [52, 53, 57, 66], guidelines on phenomenological research [56], thematic analysis [58, 59, 61], process coding [60], comparative analysis [54] and Merriam and Tisdfell’s [77] method for data analysis using open and analytical coding [55]. Some studies used theoretical frameworks to support the analysis, such as ambiguous loss theory and the model of minority stress [52], generativity theory with a critical feminist perspective and queer theory [65], the Model of Global and Situational Meaning [57], the Anderson’s Health Care Utilization Model [61] and the Resilience properties in Aging Framework [62]. Quantitative studies applied methods of analysis correspondent to the research designs, such as t-tests, chi-square test, correlations tests, linear and multivariate logistic regression among others.

Most studies reported the participant’s demographics, including ethnicity, age, gender identity, and sexual orientation, while education and income were less commonly reported. In terms of demographics, a variety of terms were used such as gender identity, gender, sex, sexual orientation and sexuality. When reporting gender identities besides male ((cis-)man) or female ((cis-)woman) some papers used terms such as transgender [46, 59, 60, 65, 68, 69], trans [66], transwoman and transman [45], while others additionally reported a variety of identities such as female with trans histories, pangendered, queer femme and trans, and male to female and back [54, 58]. To avoid misrepresenting or misinterpreting the original authors, the terminology used in the data extraction table is what is reported within the papers.

In those studies that reported ethnicity, the majority of participants were white and/or white non-Hispanic ethnicities, and the mean age varied from 52.5 to 67 years. While six of the quantitative studies mention gender identities besides men and women, five studies [43, 44, 51, 71, 75] focused exclusively on gay and bisexual men but none of the studies specifically state if all identified as cisgender. Six of the quantitative studies reported including people who identified as transgender or were reported as ‘other’ than female or male [10, 45, 46, 48, 68, 69]. These constituted between 0.6% [48] to 39.8% [45] of the sample. While Fredriksen-Goldsen et al in one paper [48] reported that 0.61% of their sample identified as other than female or male in earlier papers on the same study sample [68, 69] they report on a sample of 4.1% and 7% of transgender people. One paper by Fredriksen-Goldsen et al. [68] focused specifically on transgender people, while two studies reported included LGBT couples and spouses [52, 62], but only one [52] reported gender identity.

The predominant sexual orientation reported was gay (men), although one qualitative study focused only on lesbian women [56], and some studies focused on people who identified as bisexual [48, 54] or bisexual individuals constituted most of the sexual minority sample [50]. Only two studies reported queer identities in their sample [54, 60].

### Definition of resilience

Eleven of the papers offered definitions of resilience as either a trait, a process or an outcome [43, 46, 47, 52, 53, 55, 57, 59, 62, 69, 71]. However, there is no uniformity in the definitions offered. In the remainder of the papers, resilience was not clearly defined although it was alluded to and perceived as a strength or a positive attribute generally [10, 44, 45, 48–51, 54, 56, 58, 60, 61, 65, 66, 68, 75]. Where resilience was defined as a process, it was mainly seen as a process of adaptation where older LGBT+ individuals responded to adversities over time. Adversities were perceived as the context within which LGBT+ resilience was operationalised and in some of the papers being a member of the LGBT+ community was associated with adversity in itself. Handlovsky et al. [53], writing about gay men’s exposure to systemic discrimination, describe resilience as a response to risk exposure where environmental harms are mediated through internal and external protective processes. This allows older LGBT+ individuals to cope with adversity using both internal and external processes. King and Richardson [71] also refer to resilience as a coping mechanism, but refer to its health-promoting capacity as well its capacity to buffer physical and psychological distress. Nelson-Becker and Thomas [62] viewed resilience as ‘the capacity to manage significant difficulty and stress and is both a process and an outcome’ (p. 2), while also describing it as a character trait. As a trait, it is seen as the capacity to manage stress which is facilitated by personality characteristics and environmental supports which mirror Handlovsky et al’s. [53] definition. However, some definitions include outcome and process elements, such as Nelson-Becker and Thomas [62], who define resilience ‘as an ability to integrate life learning and expand coping repertoires, reaching a new understanding that encompasses what came before but also moves beyond it’ (p. 2) and as ‘the ability to access one’s inner wisdom and strength enhanced by time and experience’ (p. 2). Both Dziengel [52] and Nelson-Becker and Thomas [62] also bring in the notion of adversity shifting older LGBT+ individuals off-balance with resilience providing the capacity to help them to restore equilibrium.

Foster et al. [57] also define resilience in the context of it being a trait which is the capacity to respond and successfully negotiate stressful life events in addition to it being a set of protective factors. Foster et al. [57] incorporate the ability of resilience to build capacity to also respond to future challenges which stresses the developmental nature of resilience. Higgins et al. [59] refer to Foster et al’s [57] definition of resilience as well as using Ungar’s [14] trait and process orientated definition which states that resilience is the capacity to navigate resources that build and sustain wellbeing. Fleishman et al. [46] use the American Psychological Associations definition of resilience as a process of adaptation to adversity, while Fredriksen-Goldsen et al. [47, 69] discuss resilience in the context of behavioural patterns, functional competence and cultural competence that individuals and communities use in stressful situations. Emlet et al. [43] refer to resilience as a resource as well as a process, outcome and trait.

Six qualitative papers (classified as being outcome-focused) did not set out to research resilience, therefore a conceptualisation or definition of resilience was not required at the outset of these studies. In one example, the researcher referred to resilience emerging only briefly in participants’ narrations in relation to how historical experiences led the participants developing resilience in later life [66], while other authors included resilience in the discussion section of their papers [54, 56, 58, 60, 66]. In these papers, the reference to resilience is most often related to the experiences of discrimination, oppression and surviving the HIV/AIDS epidemic. These lived experiences led LGBT+ older adults to develop coping skills, which leads to resilience in later life. For example, Jen and Jones [54] relate resilience to the broader cultural and historical events and intersectional identities evident within the participants’ narratives while also recognising resilient communities’ efforts around HIV/AIDS. Green and Wheeler [61] discuss how the participants in their study demonstrated resilience as they aged which acted as a buffer to negative health care experiences related to their minority sexual orientation and HIV status. Others only briefly mention resilience as part of participants’ strengths [56, 60] and as a potential topic for training that would shift practices from pathology to seeking indicators of resiliency [58]. Only Rowan and Butler [56] set out to define *resiliency*. They define it by using Blundo’s [78] understanding of resiliency as ‘finding a stronger and more meaningful way to deal with life difficulties and stressors’ (p. 191).

### How resilience was studied

Adopting a clear definition appeared to be influenced by how the researchers approached the study of resilience. Twenty-one (n = 21) studies were classified as entry point studies as they had resilience as a stated or inferred objective and six studies were outcome studies as resilience inductively emerged from the data. In all the quantitative studies (n = 14), [10, 43–51, 68, 69, 71, 75] resilience was an entry point, while the qualitative studies, were divided between entry (n = 7), [52, 53, 55, 57, 59, 62, 65] and outcome studies (n = 6), [54, 56, 58, 60, 61, 66]. Entry point studies were further classified based on the focus researchers gave to studying resilience. Studies that utilised resilience theory and aimed to explicitly research resilience were classified as primary-focused. These studies specifically explored resilience in terms of how it was developed and the factors that influenced it. Where resilience was a secondary focus or a secondary research objective, resilience per se was researched in the context of other phenomena, such as multiple stigmas in relation to LGBTQ aging and cultural generativity [65], integration of sexual orientation and faith [57], physical and mental health [45, 50], as one of the several mental health and well-being indicators [49], and others.

Two primary focused studies specifically researched spiritual resilience [57, 62], where the focus was on the struggles LGBT people had in reconciling their sexuality with their sense of spirituality or how the persons spiritual beliefs or practices supported them in addressing life’s challenges. Others examined more general protective processes or factors that foster positive health outcomes and decreased psychological distress associated with sexual identities [53]. Resilience was also studied as an adaptive factor [44] that helps older LGBT+ people to mediate the impact of stigma and discrimination and adapt to prejudice and loss [59]. As a primary focus, resilience was also studied alongside other predictors of mental distress in gay men such as stigma, discrimination and internalised homophobia [71] and as a predictor of sexual satisfaction [46]. Resilience, along with the concept of mastery, were studied as two separate psychological resources that relate to each other but may be influenced by different factors in the structural-environmental and individual contexts [43]. In addition, Haldane et al. [55], set out to study how prominent ‘sexual orientation minority elders’ perceived the LGBTQ+ community as developing hope and resiliency in relation to major events that led to the development of rights for LGBTQ+ people.

In papers where resilience was a secondary focus, it was often seen as something that was exercised or demonstrated in the face of adversities. In Bower et al’s [65] study for example, they set out to research multiple stigmas and cultural generativity in relation to LGBTQ and ageing. They also set out a secondary objective to discuss how the experience of stigma and discrimination support a generational legacy of resilience. Similarly, Foster et al. [57] explored the integration of sexual orientation and faith and discussed how spirituality may become a method for meaning-making and resilience. Resilience as a secondary focus was more prominent in quantitative papers and often researched in relation to the presence of different physical and mental health characteristics, which were used as proxy measures of resilience. Cortes et al. [45], for example, set out to find and confirm evidence of resilience in older LGBT adults by measuring levels of anxiety, depression and alcohol use and then comparing them with younger LGBT adults. Similarly, Monin et al. [50] examined whether sexual minority status confers vulnerability or resiliency in older adulthood by using a variety of scales to measure mental health, social support and exposure to trauma. Included papers from the ‘Caring and Aging with Pride’ and ‘Aging with Pride’ studies, in most cases did not report measuring resilience specifically, but used a variety of tools to measure key health indicators, risk and protective factors and health outcomes. In these studies, higher scores in the variables measured suggest the exercise of resilience. Emlet et al. [43, 75] also drew their findings from ‘Aging with Pride’ but differed because they reported directly measuring resilience.

### Tools and conceptual frameworks used

In the studies by Batista and Pereira [44] and Pereira and Silva [51], resilience was a primary focus and was measured using the 10-item Connor-Davidson Resilience Scale that utilises a four-point Likert scale (CDRISC-10, [79]). This self-reporting scale takes the perspective that resilience is a personal quality that reflects the ability to cope with stress [80]. Fleishman et al. [46] and King and Richardson [71] used Wagnild and Young’s Resilience Scale [72], although the former used a 25-item scale, the latter used a 14-item scale without a clear rationale for omitting some of the items on the original scale. Wagnild and Young’s [72] self-reporting scale views resilience as a positive personal characteristic that enhances individual adaptation. Emlet et al. [43, 75] used a three-item scale adapted by Fredriksen-Goldsen and Kim [73] from the Brief Resilience Scale (BRS–six item) by Smith et. al [74]. The BRS was designed to assess the person’s ability to bounce back or recover from stress [74] and was used in its original form in the study conducted by Lyons et al. [49].

A number of studies used a conceptual framework to guide the research design or the analytical process. Fredriksen-Goldsen et al. [47] used a Resilience Conceptual Framework that incorporated risk and protective factors. The framework included five components: ‘(1) background characteristic (sexual orientation, gender, race etc); (2) key health indicators (access to health care, health behaviours); (3) risk factors (victimisation, stigma, concealment); (4) protective factors (social support, network size); and (5) health outcomes (general health, disability, depression)’ [47, p. 665]. While this framework was used to inform a number of the ‘Caring and Aging with Pride’ studies [47, 68, 69], there was variation in the title given to the framework and in how the dimensions of the framework were reported within the different papers published from this study.

Fredriksen-Goldsen et al. [10, 48] and Emlet et al. [75] used the Health Equity Promotion Model [70] that situates health more broadly within the life course, asserting that optimal ageing is linked to the availability of opportunities over time to promote health. ‘This model highlights how (a) social positions and (b) structural and environmental contexts intersect across the life course with (c) health-promoting and risk processes (psychological, social, behavioural, and biological) to culminate in the health and well-being of LGBT adults as they age’ [10, p. S73]. The authors argue that the key variables in the model influence resilience, therefore higher scores in health behaviours suggest the exercise of resilience. Finally, Foster et al. [57] adapted the Model of Global and Situational Meaning [81] to develop a theoretical model for Lesbian and Gay Christian Spiritual Resilience [57].

## Discussion

Of the 27 papers included in this review, the majority (n = 16) did not provide a formal definition or operationalisation of resilience at the beginning of the study. In these papers, resilience is either briefly mentioned and/or related to other concepts, which leaves the reader to deduce how researchers conceptualised resilience from the methodology used. Papers that utilised conceptual frameworks often did not provide a definition of resilience but identified it as part of the larger framework. Furthermore, considering both the papers in which resilience was an outcome of the research and papers in which resilience was the secondary focus, only ten of the 27 papers definitively set out to research resilience as a primary focus. This points to resilience being rarely researched in the older LGBT+ population as a concept in itself. This review also shows that resilience is indeed difficult to categorise as either a trait, process or an outcome, because of the fluid nature and the interrelationship between all three aspects of resilience. Therefore, researchers often write that they are researching resilience as a trait (and as a positive attribute and strength) but discuss the findings as an outcome of a lifetime of responding to adversities through a process of adaptation. Though a clear delineation might be unnecessary, there is a lack of clarity on what view of resilience is taken by some researchers and how their understanding of resilience informs their study and its methodology. Lack of clarity is further hampered by authors using other words when talking about resilience, sometimes interchangeably, words such as resiliency [e.g. 50, 52, 55], coping strategies, adaptive factor, and strengths.

Although some of the quantitative studies used scales to measure resilience, some operationalised the concept of resilience by measuring temporal changes in mental distress or mental health outcomes such as depression, anxiety and substance misuse [45] where resilience is equated with lower scores of distress or mental illness. Equating resilience with the absence of, or, reduced ‘symptomatology’ has been critiqued from the perspective of perpetuating negative perceptions of LGBT+ people as ‘at risk’ and ‘vulnerable’ as well as failing to acknowledge the wider socio/structural and system factors that impact LGBT+ lives [82, 83]. Although the study of resilience is seen as one way to shift the agenda from a risk-deficit focused approach to a more strengths-based approach [83, 84], most of the literature on resilience in the papers reviewed, discuss the specific circumstances and histories of LGBT+ older adults and research or describe resilience as a trait LGBT+ older adults possess to help them bounce back during adversity.

More importantly, the findings highlight how resilience theory is insufficiently applied throughout all stages of the research process. In keeping with previous commentary on the use of theory in research [33, 34], within the studies included in this review there was less of a tendency to use resilience theory to inform older LGBT+ research. Based on Bradbury-Jones et al. [33] typology of theory use, most of the papers met the criteria of either implied theory (level 2), partially applied (level 3) or retrospectively applied theory (level 4). Retrospective application can be observed in papers that were classified as outcome studies, where resilience theory was ‘considered at the end of the study as a means of making sense of research findings’ and/or ‘introduced as an afterthought’ [33, p. 137]. According to Bradbury-Jones et al. [33] the highest level of theory use (level 5) is the consistent application of the theory throughout the research process. This level was rarely observed, except for the studies classified as primary/entry focus which set out to research resilience (with the exception of Batista and Pereira [44, 51]), provided a review of literature and applied resilience theory throughout all stages of the research process. On the other hand, secondary focus studies rarely define resilience, and in one case even set out to measure resilience, but give no attention to presenting the theory of resilience throughout any of the phases of the study [49].

The findings also highlight, that many of the papers position their research specifically in the context of the history and effects of the HIV/AIDS pandemic on gay and bisexual men. While important, this overshadows the wider social and political landscapes that shaped gay and bisexual men’s lives, shifting the focus away from lesbian’s, bisexual women’s and transgender people’s experiences, as well as those of older LGBT+ people from Black, Asian and other ethnic minority backgrounds. Furthermore, queer identities rarely constituted a part of the sample [54, 60]. This may be due in part to the age of the study participants, as they may have viewed the terms queer as a derogatory and insulting, as opposed to a term being reclaimed by the younger activist community.

### Strengths and limitations of the review

A member of the team with specific expertise in literature searching and retrieval conducted the database searches without any time limitations being applied (GS). This helped to ensure that the review was as comprehensive as possible. In addition, the review process, data extraction and quality assessment were conducted by more than one author, making the process less susceptible to bias. However, many of the included studies had methodological weaknesses which may influence the quality of the findings in this review. Only peer-reviewed papers in the English language were included. Furthermore, papers that included resilience might have been excluded where resilience was not mentioned in the title/abstract/keywords section of the paper or in cases where researchers used terminology other than resilience although our search strategy sought to minimise this. While some might consider the inclusion of multiple papers from the same study a limitation, with the potential to skew the findings, their inclusion shows how even within the same study resilience is differently applied and conceptualised across the papers. Deciding on whether resilience was a primary or secondary focus had an element of subjectivity attached. For example, we classified Haldane et al. [55] study as a primary focused paper, as the study of resiliency was stated as an aim; however, as resiliency was not included in the subsequent research questions or reflected in the interview schedule others may have classified this paper differently.

### Future research

In terms of future research this review points to the need for researcher to explore in greater depth from an emic perspective how older LGBT+ people conceptualise resilience, including the socio structural factors that promote or hinder its development. It is also important that future studies are designed in a way that takes account of peoples’ multiple identities (e.g. gender, sexual orientation, ethnicity) might intersect and influence their understandings and experiences of resilience. The review demonstrates that white, gay and middle-class men were more prevalent in the samples of the included studies, therefore the question of whom is being studied needs to be considered in any future research on resilience in LGBT+ later life. In addition, future researcher should be more explicit about how they are using resilience theory, including the discourse or perspective they are taking when employing the concept. As highlighted in the review more studies are also required to make explicit how concepts such as resilience, resiliency, coping and adaptation differ. If the study of resilience is to move towards a strengths-based approach, there is a need for researchers to consider their choice of measurement scales and move beyond scales that measure symptomatology. There is also a need for future resilience studies to move beyond the ‘individual’ adaptation discourse to one that addresses or politicises resilience in the context of socially embedded discrimination experienced by the older LGBT+ community. Finally, while not specifically focused on resilience, to avoid confusion there is a need for researchers to be cognisant of the language they use to report demographic characteristics, especially when reporting on sex, gender and sexual orientation.

## Conclusions

There has been an increasing growth of research into the experiences of LGBT+ ageing internationally, which has attempted to connect with more recent advances in developing theoretical models of resilience both in the LGBT+ population [26, 27] and ageing [21]. The body of research reviewed here has considered the interactional and contextual features of both ageing and LGBT+ identities sometimes without a clear definition of resilience, and the variety of studies tend to show a lack of coherence in the way in which the concept of resilience is utilised and theorised in LGBT+ ageing.

This review is the first in our knowledge to have examined how resilience is actually defined and conceptualised in LGBT+ ageing research and highlights some of the challenges in providing an adequate comprehension of resilience and how this can be better integrated. Further work to develop a typology of resilience theory taking a more ecological perspective may help to apply resilience to LGBT+ ageing research in a more comprehensive way. Understanding the mechanisms involved in resilience, including the minority stress and structural issues that impact resilience has the potential to advance our knowledge and create new and innovative theoretical approaches [85] to inform affirmative policies and practices. Furthermore, greater clarity around the concept of resilience would support the education and practice of professionals and providers in relation to resilience and unique experiences of older LGBT+ adults [32, 86, 87].

## Supporting information

S1 Appendix Example search in Medline (Ebsco). (TIF)

S1 Table Inclusion/exclusion criteria. (TIF)

S1 File (a and b). PRISMA checklist.
(ZIP)
