# Supplementary figures and images for: Defining and researching the concept of resilience in LGBT+ later life: Findings from a mixed study systematic review
Source: PLoS One. 2022 Nov 11;17(11):e0277384. doi: 10.1371/journal.pone.0277384 (PMC9651550; doi:10.1371/journal.pone.0277384)

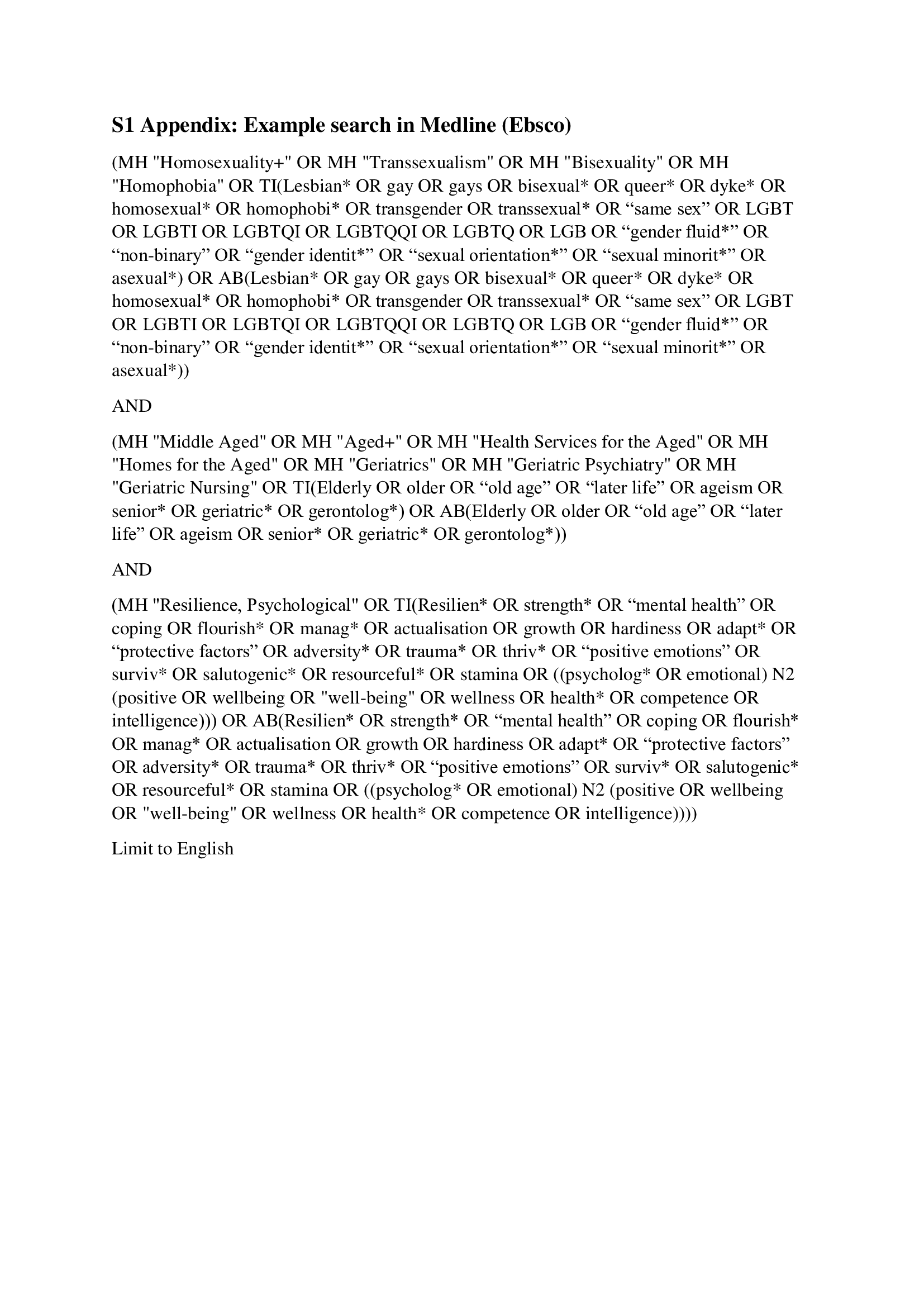

Supplement: S1 Appendix — (TIF) [file pone.0277384.s001.tif]

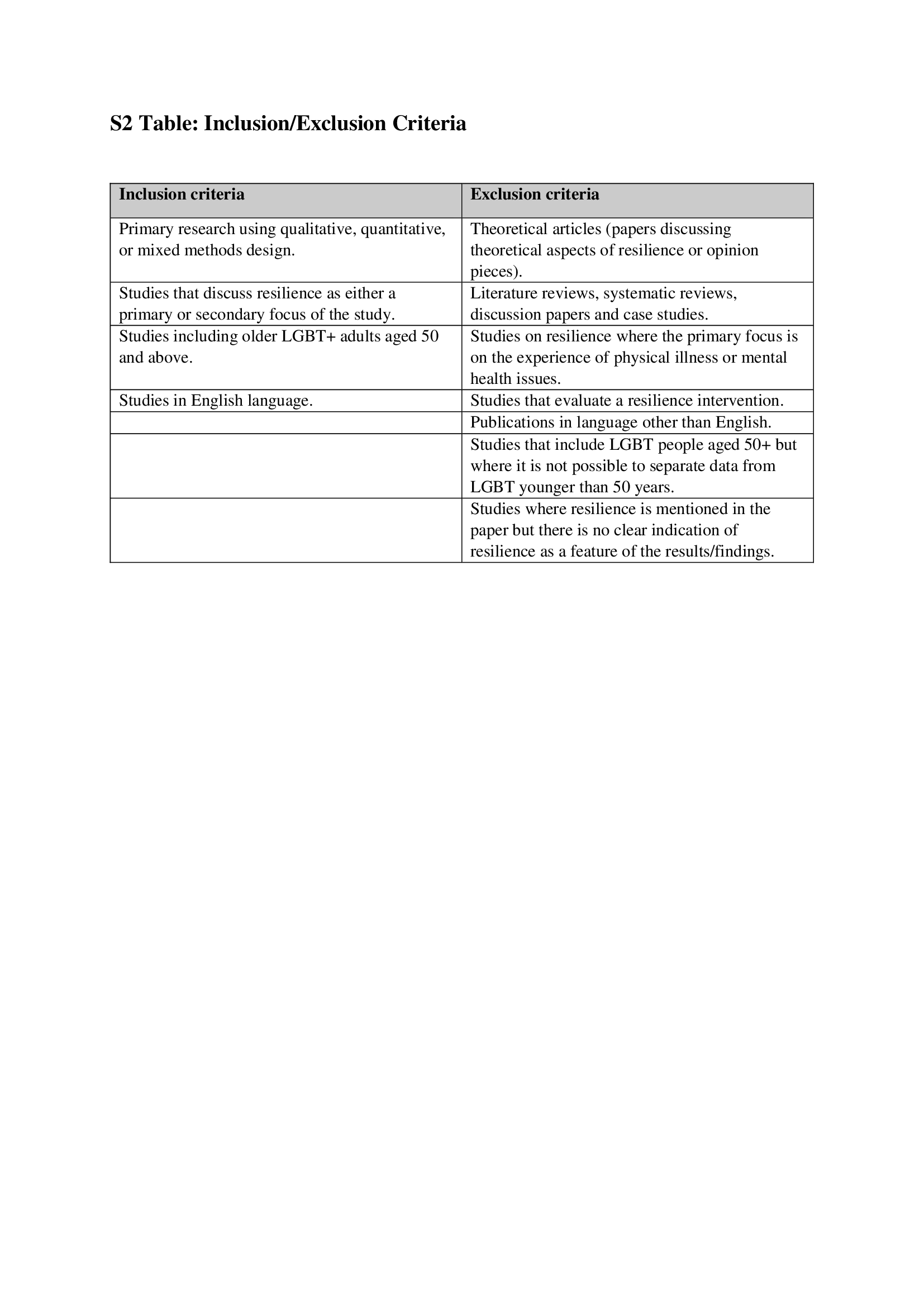

Supplement: S1 Table — (TIF) [file pone.0277384.s002.tif]

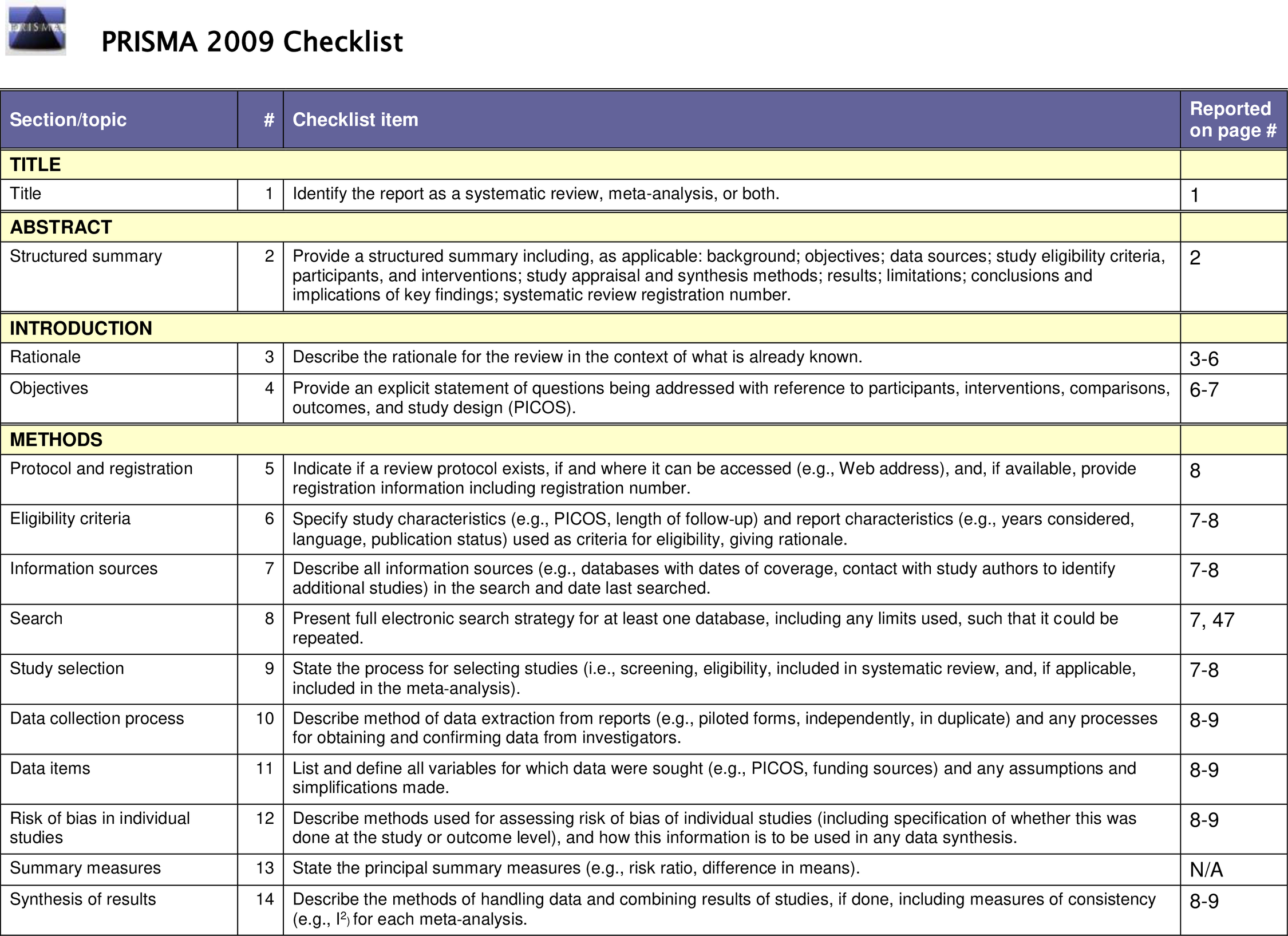

Supplement: S1 File — (a and b). PRISMA checklist. (ZIP) [file pone.0277384.s003.zip › S1a_File.tif]

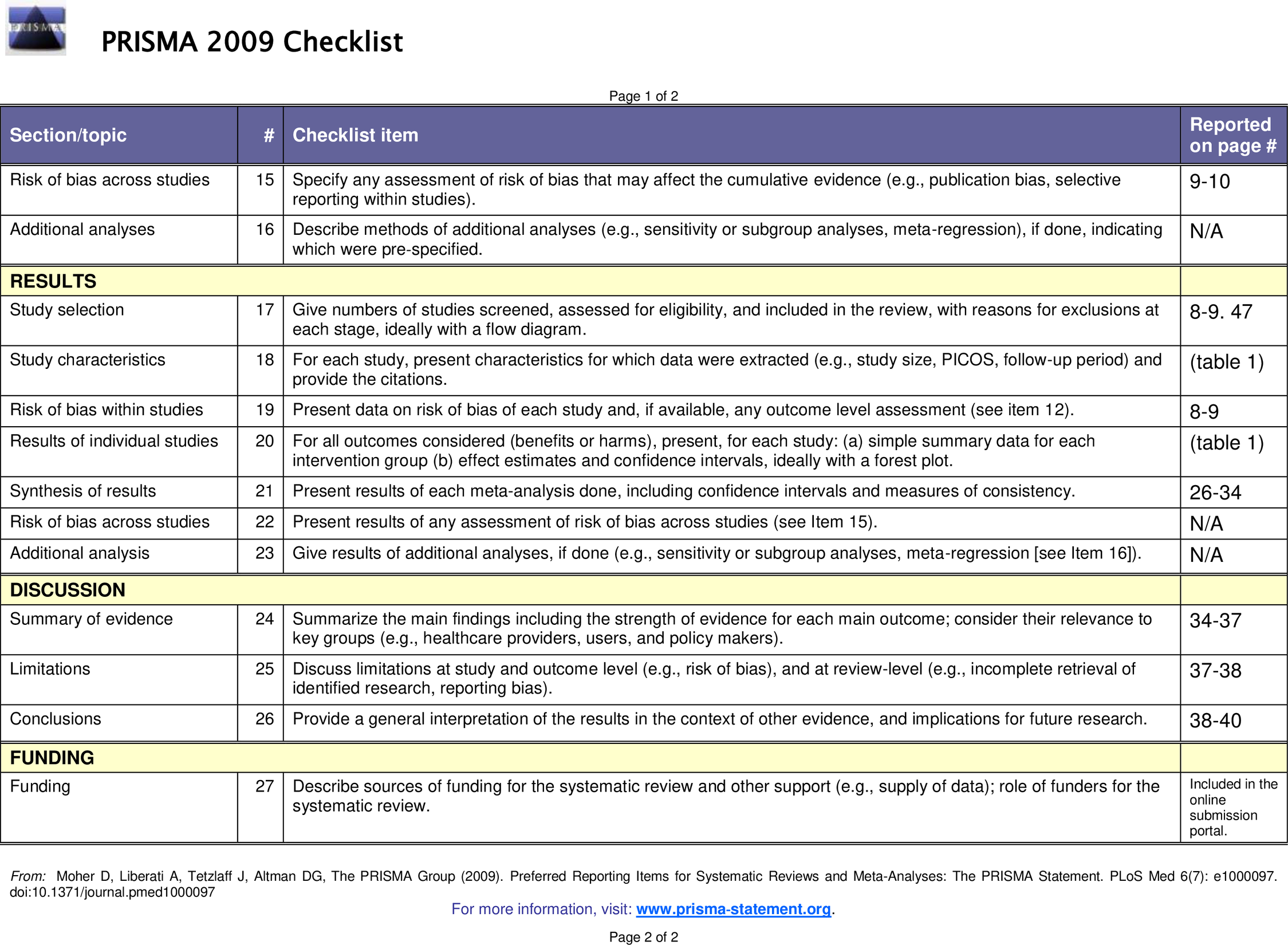

Supplement: S1 File — (a and b). PRISMA checklist. (ZIP) [file pone.0277384.s003.zip › S1b_File.tif]
